# Supplementary material for: Baseline Characterization of the Gut Microbiota of Field and Colony Populations of Phlebotomus tobbi and Preliminary Assessment of the Anti-Leishmanial Activity of Cultivable Bacteria
Source: Pathogens. 2026 Jun 23;15(7):658. doi: 10.3390/pathogens15070658 (PMC13416292; doi:10.3390/pathogens15070658)
Supplement: Supplementary file 1 [file pathogens-15-00658-s001.zip › pathogens-4357168-File S1/Sample_Information/sample_metadata.html]

| sample\_id | customer\_label | raw\_seq\_files |  |
| --- | --- | --- | --- |
| zr4724\_1V3V4 | Patient1 | zr4724\_1V3V4\_R1.fastq.gz;zr4724\_1V3V4\_R2.fastq.gz | NA |
| zr4724\_2V3V4 | Patient2 | zr4724\_2V3V4\_R1.fastq.gz;zr4724\_2V3V4\_R2.fastq.gz | NA |
| zr4724\_3V3V4 | Patient3 | zr4724\_3V3V4\_R1.fastq.gz;zr4724\_3V3V4\_R2.fastq.gz | NA |
| zr4724\_4V3V4 | Patient4 | zr4724\_4V3V4\_R1.fastq.gz;zr4724\_4V3V4\_R2.fastq.gz | NA |
| zr4724\_5V3V4 | Patient5 | zr4724\_5V3V4\_R1.fastq.gz;zr4724\_5V3V4\_R2.fastq.gz | NA |
| zr4724\_6V3V4 | Patient6 | zr4724\_6V3V4\_R1.fastq.gz;zr4724\_6V3V4\_R2.fastq.gz | NA |
| zr4724\_7V3V4 | Patient7 | zr4724\_7V3V4\_R1.fastq.gz;zr4724\_7V3V4\_R2.fastq.gz | NA |
| zr4724\_8V3V4 | Patient8 | zr4724\_8V3V4\_R1.fastq.gz;zr4724\_8V3V4\_R2.fastq.gz | NA |
| zr4724\_9V3V4 | Patient9 | zr4724\_9V3V4\_R1.fastq.gz;zr4724\_9V3V4\_R2.fastq.gz | NA |
| zr4724\_10V3V4 | Patient10 | zr4724\_10V3V4\_R1.fastq.gz;zr4724\_10V3V4\_R2.fastq.gz | NA |
